# Hybrid Plasticizers Enhance Specificity and Sensitivity of an Electrochemical-Based Sensor for Cadmium Detection

**DOI:** 10.3390/ijms23126402

**Published:** 2022-06-08

**Authors:** Pattarawan Ruangsuj, Suwanan Wanthongcharoen, Woraphan Chaisriratanakul, Win Bunjongpru, Wariya Yamprayoonswat, Wutthinan Jeamsaksiri, Watthanachai Jumpathong, Montri Yasawong

**Affiliations:** 1 Program on Environmental Toxicology, Chulabhorn Graduate Institute, Chulabhorn Royal Academy, Bangkok 10210, Thailand; pattarawan@cgi.ac.th (P.R.); suwanan@cgi.ac.th (S.W.); 2 Thai Microelectronics Center, National Science and Technology Development Agency (NSTDA), Chachoengsao 24000, Thailand; woraphan.chaisriratanakul@nectec.or.th (W.C.); win.bunjongpru@nectec.or.th (W.B.); wutthinan.jeamsaksiri@nectec.or.th (W.J.); 3 Digital Agriculture Technology Research Team, Deputy Executive Director Research and Development Intelligent System and Networks, National Electronics and Computer Technology Center, National Science and Technology Development Agency, Pathum Thani 12120, Thailand; wariya.yam@ncr.nstda.or.th; 4 Program on Chemical Sciences, Chulabhorn Graduate Institute, Chulabhorn Royal Academy, Bangkok 10210, Thailand; 5 Center of Excellence on Environmental Health and Toxicology (EHT), Office of Higher Education, Bangkok 10210, Thailand

**Keywords:** cadmium(II), hybrid plasticizers, cadmium ionophore, ion transfer, heavy metals, electrochemical sensor

## Abstract

In addition to their use as an additive to improve physical properties of solvent polymeric membranes, plasticizers have a considerable impact on the specificity and sensitivity of membrane-modified electrochemical sensors. In this work, we aim at the hybridization of two different plasticizers using the electropolymerization technique in the development of a cadmium(II)-selective electrochemical sensor based on screen-printed gold electrode along with cyclic voltammetric measurement. At this point, we first screen for the primary plasticizer yielding the highest signal using cyclic voltammetry followed by pairing it with the secondary plasticizers giving rise to the most sensitive current response. The results show that the hybridization of DOS and TOTM with 3:1 weight ratio (~137.7-μm-thick membrane) renders a signal that is >26% higher than that from the sensor plasticized by DOS per se in water. The solution of 0.1 mM hydrochloric acid (pH 4) is the optimal supporting electrolyte. In addition, hybrid plasticizers have adequate redox capacity to induce cadmium(II) transfer from bulk solution to the membrane/water interfaces. Conversion of voltammetric signals to semi-integral currents results in linearity with cadmium(II) concentration, indicating the irreversible cadmium(II) transfer to the membrane. The DOS:TOTM hybrid sensor also exhibits high sensitivity, with a limit of detection (LOD) and limit of quantitation (LOQ) of 95 ppb and 288 ppb, respectively, as well as greater specificity towards cadmium(II) than that obtained from the single plasticizer sensor. Furthermore, recovery rates of spiked cadmium(II) in water samples were higher than 97% using the hybrid plasticizer sensor. Unprecedentedly, our work reports that the hybridization of plasticizers serves as ion-to-electron transducer that can improve the sensor performance in cadmium(II) detection.

## 1. Introduction

Ion-selective electrodes (ISEs) are constantly being developed for several areas of research and clinical application. Conventionally, ISEs are used as sensors in potentiometric measurements (zero-current approach), where the response is derived from the equilibrium potential between ISEs and the reference electrode [1,2]. The analytical challenges posed by potentiometric ISEs include the irreproducible potential drift at the membrane/electrode interface caused by the prevention of charge transfer at the ISE interface and the metallic electrode. To acquire a stable response, potentiometric ISEs are essentially fabricated with appropriate plasticizers and/or conductive polymers to allow the flow of charges at the ISE interface and to enhance the analytical performance of potentiometric ISEs.

Conversely, potential energy applied to voltammetric ISEs (nonzero-current approach) leads to polarizable membrane/water interface (immiscible electrolyte solutions, ITIES) [3,4,5]. This polarizable membrane drives ions from the bulk water phase to the other, which contributes to much higher current response than potentiometric measurements. Ion transfer voltammetry is typically performed by cyclic voltammetry along with linear sweep potential that provides us with a mechanistic understanding of the ion transfer process based on the current–potential relationship. Unlike potentiometry, voltammetric ISEs can determine two or more ions simultaneously, as long as the half-wave potentials of the ion transfer reaction are well-separated.

Here, we report the enhanced sensitivity of using hybrid plasticizers on a cadmium(II) ionophore-based voltammetric screen-printed gold electrode (SPGE) supported by PVC membrane. It is noted that hybrid materials present variability in terms of the appearance and arrangement of their materials. The formation of hybrid materials is based more on chemical interactions than on physical interactions [6]. Cadmium(II) is selected as the analyte in this work, because this element is deleterious to human health despite presenting at very low levels. At this point, we first acquire the primary plasticizer derived from screening through ten different types of plasticizing compounds using cyclic voltammetry to measure the current signals. Then, reiterating the mentioned process by adding another plasticizer provides the optimized hybrid plasticizer-based sensor. Finally, the performance of the hybrid-plasticized sensor, including sensitivity and specificity, is determined in cadmium(II)-spiked water samples.

## 2. Results and Discussion

**Figure 1:**
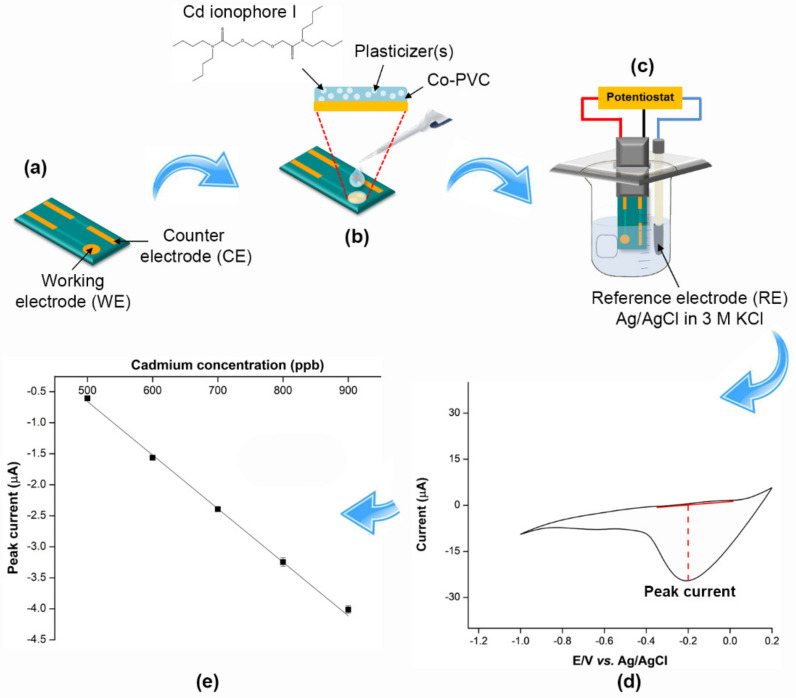
SPGE-based cadmium sensor development and voltammetric measurement. (**a**) SPGE containing working electrode (WE) and counter electrode (CE), (**b**) gold surface of working electrode was modified with membrane solution composed of co-PVC, plasticizers and cadmium ionophore I for cadmium(II) detection, (**c**) modified SPGE was connected to potentiostat and external referent electrode (Ag/AgCl in 3 M KCl), (**d**) the voltammogram representing the peak current of the sensor, and (**e**) linear current response observed from peak current of voltammogram.

**Figure 2:**
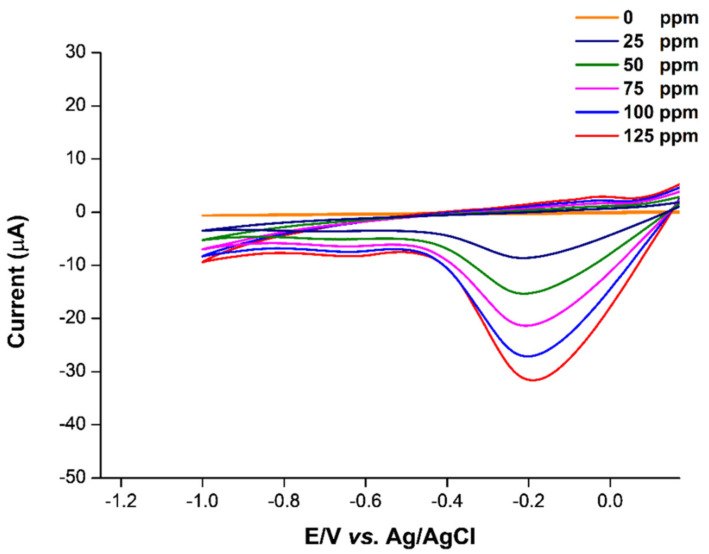
Voltammogram of the single plasticizer sensor. Peak current of the sensor is represented at the potential of −0.18 V.

The preparation of a single/hybrid plasticized PVC-membrane and the fabrication of the membrane on SPGE to construct the cadmium-selective electrode is illustrated in Figure 1. The plasticizers play a crucial role in the selectivity [7], sensitivity [8] and mechanical properties [9] of the sensor. Therefore, the types and amounts of the plasticizers are screened and optimized to obtain the ion-selective electrode (ISE) with high analytical performance. In this work, the addition of the suitable secondary plasticizer to the primarily plasticized PVC membrane contributes to the enhancement of sensitivity and selectivity in cadmium(II) detection in the voltammetric measurement. Subjected to linear-sweep cyclic voltammetry in the presence of different cadmium(II) concentrations, the fabricated sensor only plasticized by DOS shows a negatively peak current of cadmium(II) (Figure 2). This indicates that cadmium(II) is transferred and bound to the ion-selective membrane, suggesting that the ion-selective PVC membrane is physically well-adsorbed on the SPGE after being immersed into water during the electroanalytical analysis.

### 2.1. Screening for the Primary Plasticizer

**Figure 3:**
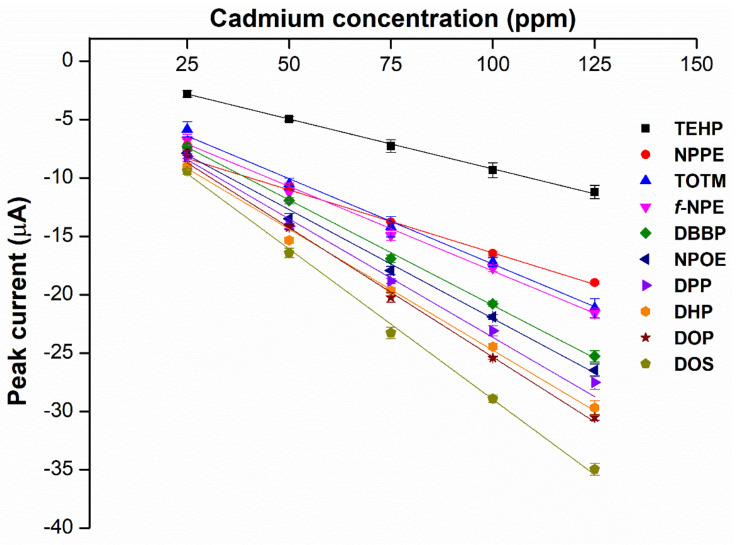
Relationship between peak current of the different types of primary plasticizer sensors and the different concentrations of cadmium(II), (*n* = 5).

To explore which primary plasticizer can give the highest current response (Table 1), ten different plasticizers are selected based on the three criteria: degree of linearity, molecular weight, and dielectric constant. These properties greatly influence the performance of the cadmium ISE [10]. To this end, ten types of cadmium-selective PVC membrane based on different plasticizers are added on the SPGE and subjected to electropolymerization using cyclic voltammetric technique. Intriguingly, SPGEs modified by the plasticizers of low dielectric constants including DOS, DOP and DHP exhibit the top three highest current responses (Figure 3), rather than the NPPE and *f*-NPE-modified sensors, whose dielectric constants for those two plasticizers are the highest among the others (Table 1). This result strongly suggests that dielectric constant values are overridden by other factors, e.g., the molecular geometry of the plasticizers.

In this work, DOS is the most suitable plasticizer in the fabrication of the sensor. Our result suggest that DOS recruits the highest concentration of cadmium ionophore among the others, resulting in the highest peak current. Additionally, due to the lipophilicity of the ionophore, leakage to water is negligible. Furthermore, we discuss that the structure of DOS containing -CH_2_- single-bonded connections increases the membrane flexibility to enrich the cadmium ionophore I at higher concentration, which stems from the extensive interaction between those alkyl groups of DOS and PVC. Therefore, DOS was selected as a primary plasticizer for developing the hybrid plasticizer sensor in cadmium(II) detection.

**Table 1:** Chemical properties of plasticizers used for the construction of cadmium-selective membrane; Ɛ = dielectric constant.

No.	Plasticizer	Abbr.	Structure	Type	M.W.	Ɛ	Reference
1	2-fluorophenyl 2-nitrophenyl ether	*f*-NPE	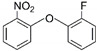	Ether	233.20	50.0	[11]
2	2-Nitrophenyl phenyl ether	NPPE	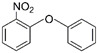	Ether	215.20	24.0	[12]
3	2-Nitrophenyl octyl ether	NPOE	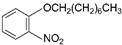	Ether	251.32	24.0	[13]
4	Tris(2-ethylhexyl) phos-phate	TEHP	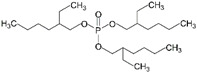	Phosphoric acid ester	434.64	4.8	[14]
5	Dibutyl butylphosphonate	DBBP	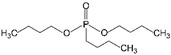	Phosphoric acid ester	250.31	4.6	[14]
6	Dioctyl phthalate	DOP	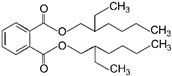	Phthalate ester	390.56	5.0	[14]
7	Diheptyl phthalate	DHP	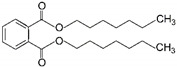	Phthalate ester	362.50	~4.0–9.0	[15]
8	Diphenyl phthalate	DPP	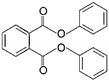	Phthalate ester	318.32	~4.0–9.0	[15]
9	Tris(2-ethylhexyl) trimelli-tate	TOTM	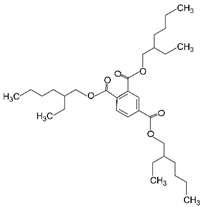	Trimellitate ester	546.79	4.9	[16]
10	Bis(2-ethylhexyl) sebacate	DOS	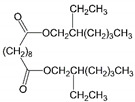	Dibasic acid esters	426.67	4.0	[13]

### 2.2. Effects of Hybrid Plasticizers on Cadmium Detection 

**Figure 4:**
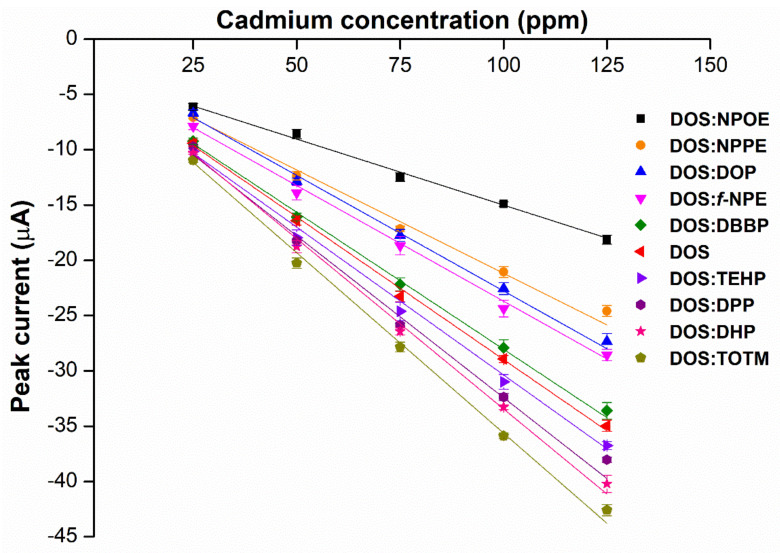
Relationship between peak current of the different types of hybrid plasticizer sensors and the different concentrations of cadmium(II), (*n* = 5).

Following a priori knowledge [17], a series of the hybrid plasticizers is firstly fabricated with the weight ratio of 5:1 (DOS:the others) to attain average molecular weights of >400 Da (Table 2). Among the other hybrid-plasticized sensors (Figure 4), DOS:TOTM and DOS:DHP are the two sensors that exhibit the highest current responses with a significant difference (*p*-value < 0.05). In addition, the current responses measured from those two hybrid-plasticized sensors are 26% and 19%, respectively, higher than that obtained from the DOS-plasticized sensor per se. Our results indicate that the hybrids of different plasticizers can enhance the sensing performance of PVC-based sensors.

To our surprise, the use of TOTM as a secondary plasticizer can boost the sensitivity of the DOS-plasticized sensor, given the low current response from the first screening (Figure 3). We hypothesize that the aromatic residues in TOTM and DHP enhance electron delocalization between the gold surface and the membrane. In addition, the bulky structure of TOTM allows a higher concentration of cadmium ionophore I to accumulate in the membrane. Therefore, the DOS:TOTM hybrid-plasticized sensor is selected for further investigation of the optimal weight ratio of the hybrid plasticizers.

**Table 2:** The average molecular weight of the hybrid plasticizers.

No.	Hybrid plasticizers (5:1)		Average M.W.
	Plasticizer A	Plasticizer B	
1	DOS	NPOE	397.45
2	DOS	NPPE	391.43
3	DOS	DOP	420.65
4	DOS	*f*-NPE	394.43
5	DOS	DBBP	397.28
6	DOS	TEHP	428.00
7	DOS	DPP	408.61
8	DOS	TOTM	446.69
9	DOS	DHP	415.98

### 2.3. Effects of Weight Ratios of Hybrid Plasticizers on Sensor Performance

**Figure 5:**
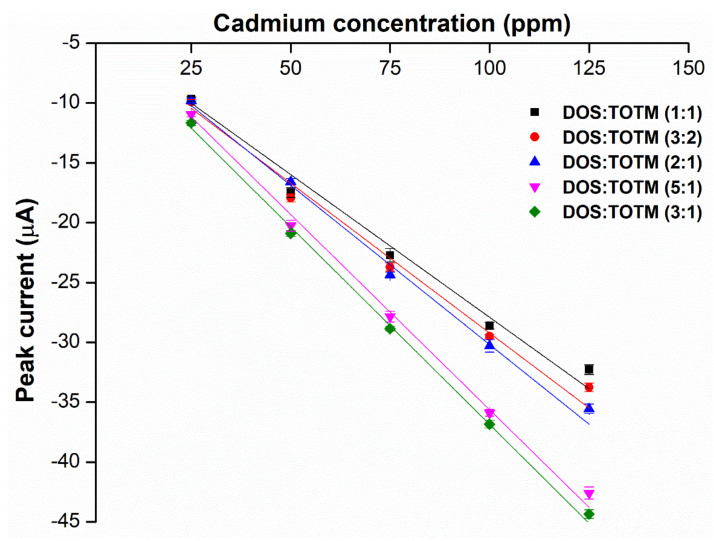
Relationship between peak current of the different weight ratios of DOS:TOTM hybrid plasticizer sensors and different concentrations of cadmium(II), (*n* = 5).

Acquiring the optimal hybrid plasticizers, we next sought the weight ratio of DOS:TOTM that is able to achieve the highest sensor sensitivity. Depicted in Figure 5, the weight ratios of 3:1 (~137.7-μm-thick membrane) and 5:1 (~136.3-μm-thick membrane) (DOS:TOTM) yield the highest current response with no significant difference (*p*-value > 0.05). However, the 3:1 hybrid sensor shows a 7% higher average peak current than the 5:1 hybrid sensor at the lowest cadmium(II) concentration of 25 ppm. The lower signals given by the 2:1 (~139.1-μm-thick membrane), 3:2 (~140.3-μm-thick membrane) and 1:1 (~142.0-μm-thick membrane) hybrid sensors are presumably a result of the lower flexibility of the polymer chain in the membrane leading to poorer mobility of cadmium ionophore I to form complex with cadmium(II). However, when the concentration of plasticizers is too high, the polymer chain in the membrane with greater flexibility allows the cadmium ionophore to non-specifically interact with other cations in the system [18] besides the coextraction with counterions [19]. Therefore, the 3:1 weight ratio of DOS:TOTM was selected for further electroanalytical analysis for cadmium(II) detection. Please note that the average molecular weight of the optimal hybrid plasticizers at the ratio of 3:1 is 456.70 g/mol, thus providing a balance between the flexibility and rigidity of the polymer chain [18].

### 2.4. Choice of Supporting Electrolytes and pH 

**Figure 6:**
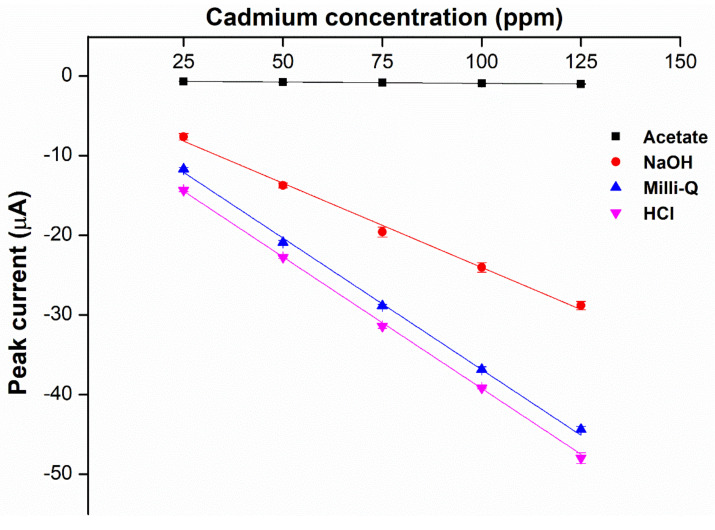
Effect of supporting electrolytes (SE) on the sensitivity of the hybrid plasticizer sensor (*n* = 5).

**Figure 7:**
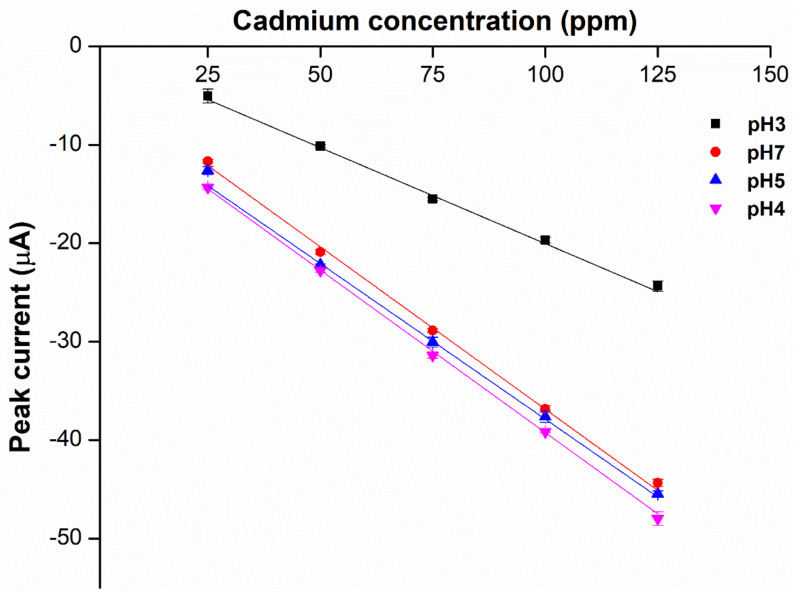
Effect of different pH using HCl on the sensitivity of the hybrid plasticizer sensor (*n* = 5).

When searching for the most suitable supporting electrolyte (SE), different pHs and salt species are the key factors affecting the current response. A range of electrolytes, including acetic acid/acetate buffer (0.1 M, pH 4.5), hydrochloric acid solution (0.1 mM, pH 4) and sodium hydroxide solution (1 µM, pH 8), have been used as solvents in cadmium(II) detection. Again, the parameter used for acquiring the most suitable electrolyte for this system is the current response (Figure 6). Among the investigated SEs, 0.1 mM hydrochloric acid (pH 4) gives the highest current response (Figure 7) in the presence of trace cadmium(II) (Figure 6). At pH 3 of the hydrochloric acid solution, the current response is lower than that at pH 4, which is derived from the interference of H^+^ with cadmium ionophore [18]. As to the very low response of cadmium(II) using acetic acid/acetate buffer (pH 4.5), the poor ionization of the weak acid gives a low concentration of ions in the system. Moreover, the current response of the hybrid plasticizer sensor is diminished at pH 8 (Figure 7), resulting from the decreased level of cadmium(II) in the solution via cadmium(II) hydrolysis [18]. At this point, hydrochloric acid of pH 4 was selected for further electroanalytical analysis for cadmium ion detection. Therefore, hydrochloric acid solution (0.1 mM, pH 4) was used for electrochemical polymerization of DOS and TOTM onto SPGEs. As shown in Figure S1, the electropolymerization of DOS and TOTM is successful, as can be observed by the decrease in the anodic current after the first cycle (five scans of the 10th cycle), which is derived from the consumption of the monomer [20]. As can be seen in Figures S1 and S2, the chemical shift of δ 8.03 ppm (singlet peak) indicates that carbon at position 4 of TOTM forms a covalent bond with DOS. However, the percent yield of polymerization is 15%, based on the integration of *^1^*H of the peaks (Figures S2 and S3). The unreacted TOTM and DOS are detected after the membrane is washed with THF using HPLC-UV (Figures S4 and S5).

### 2.5. Mechanistic Determination

**Figure 8:**
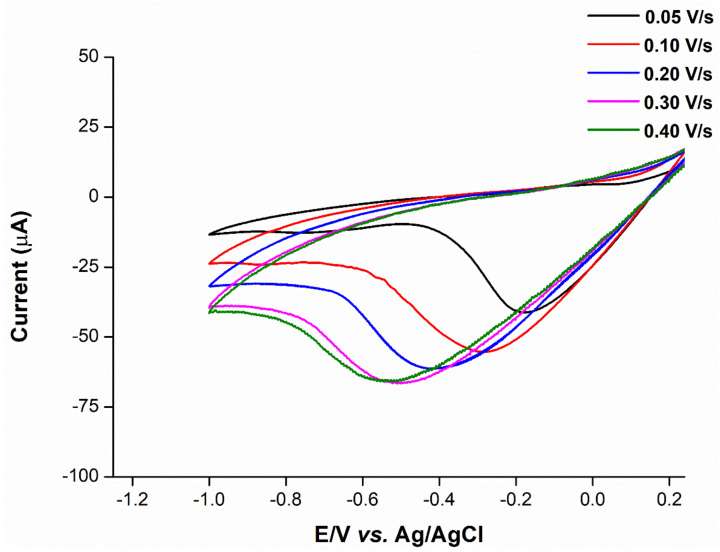
Voltammogram of the DOS:TOTM hybrid sensor in 125 ppm cadmium(II) and 0.1 mM HCl solution at different scan rates (0.05, 0.10, 0.20, 0.30 and 0.40 V/s).

**Figure 9:**
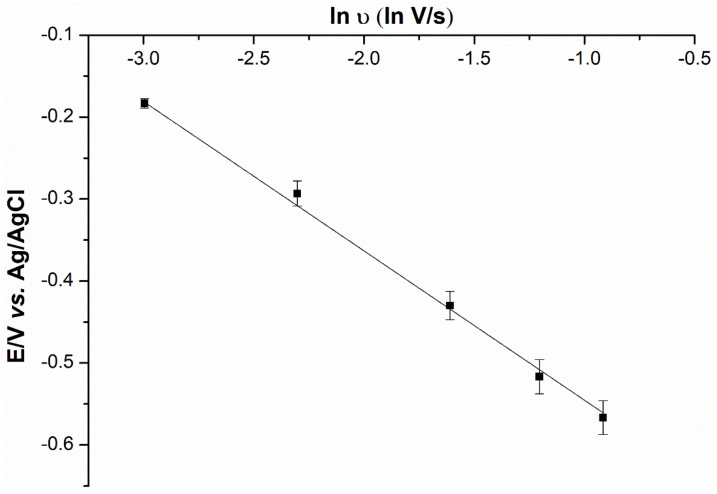
The peak potential (*E*) against the natural logarithm of scan rate (ln ʋ) for the voltammogram of 125 ppm cadmium(II) in 0.1 mM HCl solution (*n* = 5).

To determine whether the ion transfer mechanism was present on the electrode surface, scan rates ranging from 0.05 to 0.40 V/s were applied to the hybrid-plasticized SPGE, followed by the measurement of the current signals. As can be seen from the cyclic voltammogram (Figure 8), the signals increase with higher scan rates, along with the characteristics of peak-to-peak separation. Regarding the process of charge transport, the electrochemical reversibility observed from the scan rate is independent of the peak potential of analyte [21]. According to Equation (1) [22], the linearity of the plot is a result of the relationship between peak potential (*E*) and the natural logarithm of scan rate (ln ʋ) (Figure 9), which indicates the irreversibility of cadmium(II) transfer to the hybrid-plasticized ISE.

(1) $$\mathrm{Slope}=\frac{\partial E}{\partial \ln\;ʋ}=\frac{RT}{2\alpha z_{i}F}$$

The value between *E* and ln ʋ of 182 mV, obtained from Equation (1), indicates the super-Nernstian response from the voltammetric ion transfer process. The transfer coefficient of cadmium(II) transfer, calculated from Equation (1), is α = 0.034 ± 0.002, which is much less than 0.50 [23].

To gain further mechanistic insights into cadmium(II) transfer, the current signals obtained from the background-subtracted voltammograms of cadmium(II) transfer at the concentrations of 25, 50, 75, 100 and 125 ppm are converted to the semi-integral of current (*I*(*t*)) (Equation (4)) shown in Figure S6. The sigmoidal forward waves indicate the irreversibility of cadmium(II) transfer. The sigmoidal curve of each cadmium concentration provided the limiting value of the semi-integral (*I_l_*) that is proportional to the cadmium concentrations (Figure S7). In addition, the relationship of the semi-integral (*I*(*t*)) with cadmium(II) concentration can provide us with ion transfer parameters: the diffusion coefficient (*D_i_*) in the aqueous phase of cadmium(II) transfer is 0.81 × 10^−5^ cm^2^/s calculated from the slope (*I_l_*/*c_i_* = 38.9 C·cm^3^·s^−1/2^·mol^−1^) using Equation (2) [23], where the area of the working electrode (*A*) is 7.07 × 10^−2^ cm^2^.

(2) $$D_{i}={\left(\frac{I_{l}}{z_{i}AFc_{i}}\right)}^{2}$$

**Figure 10:**
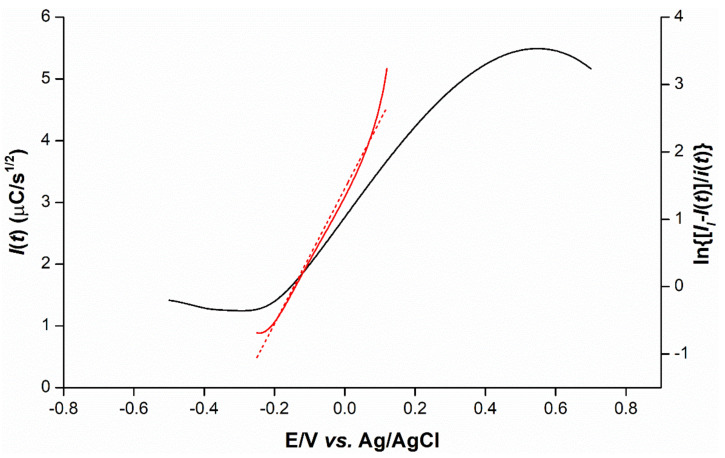
Semi-integral (*I*(*t*)) of a background-subtracted voltammogram (black line) and a plot of ln{[*I_l_*–*I*(*t*)]/*i*(*t*)} with potential (*E*) (red line). The short dashed line is the linear fit. The voltammogram derived from the DOS:TOTM hybrid sensor in 125 ppm cadmium(II) and 0.1 mM HCl solution at a scan rate 0.05 V/s.

The *D_i_* value of cadmium(II) in this study is 26% higher than that calculated in a previous study [24], meaning faster diffusion. At 125 ppm of cadmium(II), a plot between ln{[*I_l_*−*I*(*t*)]/*i*(*t*)} and applied potential (*E*) presents a linearity with the sigmoidal curve (Figure 10), where *i*(*t*) is the voltammetric peak current. Subsequently, the acquisition of rate constant of the forward reaction (*k_f_*) can be achieved by the calculation from Equation (3) [21] using the *D_i_* value and semi-integral current, yielding the *k_f_* value of 1.18 × 10^−5^. Much lower than $\sqrt{D_{i}FʋR^{-1}T^{-1}}$ [25], the *k_f_* value from our results indicates that cadmium(II) transfer to the membrane is irreversible.

(3) $$lnk_{f}=ln\sqrt{D_{i}}-ln\frac{I_{l}-I\left(t\right)}{i\left(t\right)}$$

### 2.6. Anion Interference

**Figure 11:**
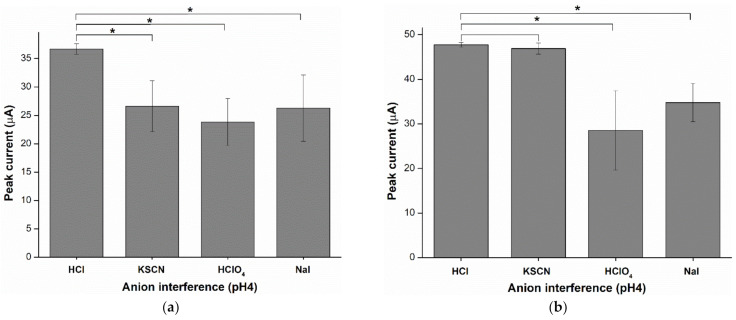

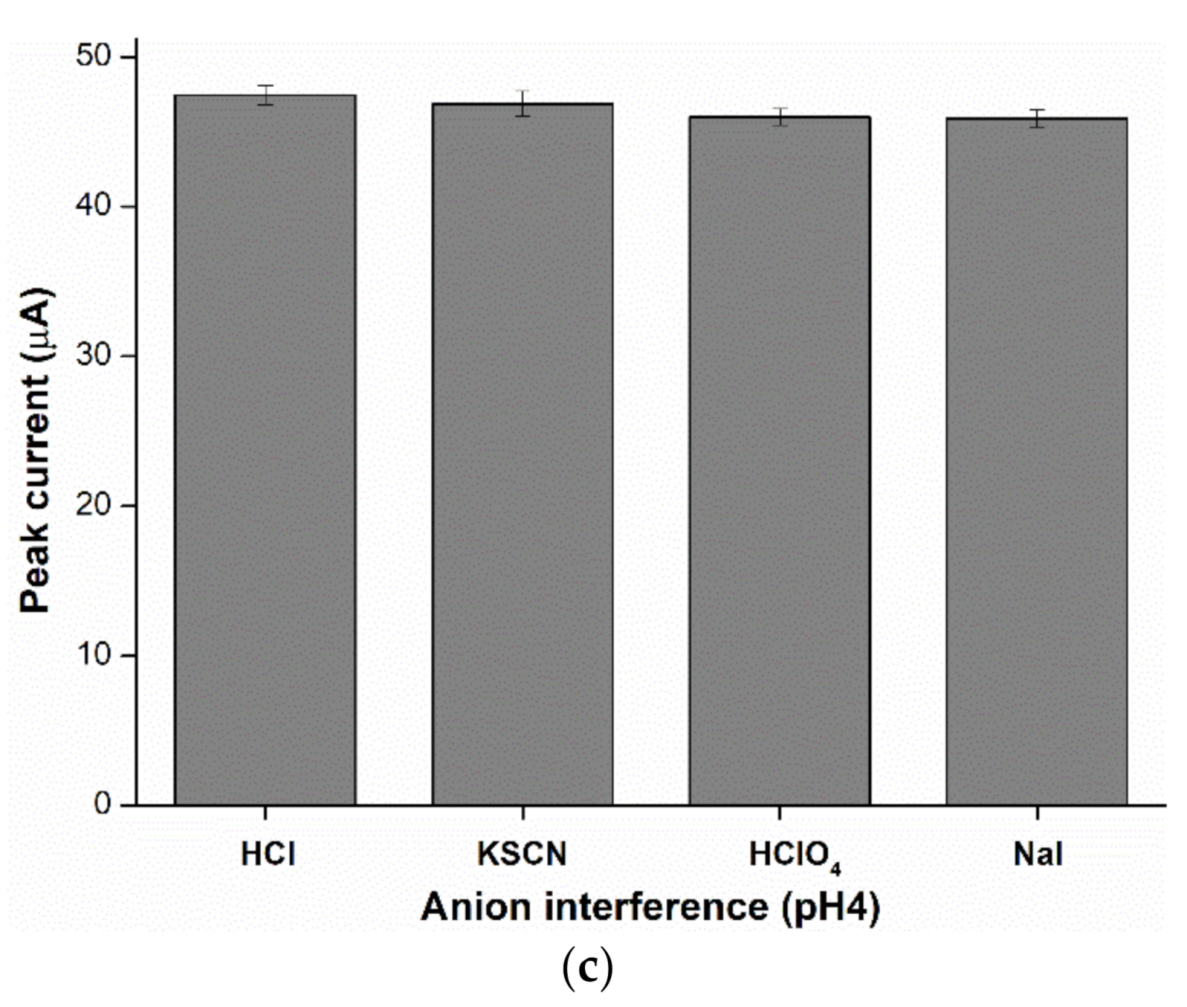
Coextraction of anion interferences affects the peak current of cadmium(II): (**a**) single (DOS) plasticizer sensor; (**b**) hybrid (DOS:TOTM) plasticizer sensor; (**c**) hybrid (DOS:TOTM) plasticizer sensor composed of TBACl (*n* = 5), (* *p*-value < 0.05).

Shown in Figure 11a, the sensor singly plasticized by DOS undergoes interference by anions including thiocyanate (SCN^−^), perchlorate (ClO_4_^−^) and iodide (I^−^), as reflected in the significant decrease in peak current. However, the hybrid plasticized sensor (DOS:TOTM) is not interfered with by SCN^−^ (Figure 11b, *p*-value > 0.05). This suggests that the hybrid plasticized sensor can improve the coextraction of cadmium(II) with thiocyanate. The hybrid plasticizer membrane composed of the ion exchanger (tetrabutylammonium chloride, TBACl) is not interfered with by these three anions (Figure 11c, *p*-value > 0.05). Therefore, TBACl is required to obtain stable cadmium(II) transfer to the membrane.

### 2.7. Specificity and Sensitivity of the Hybrid Plasticizer Sensor

**Figure 12:**
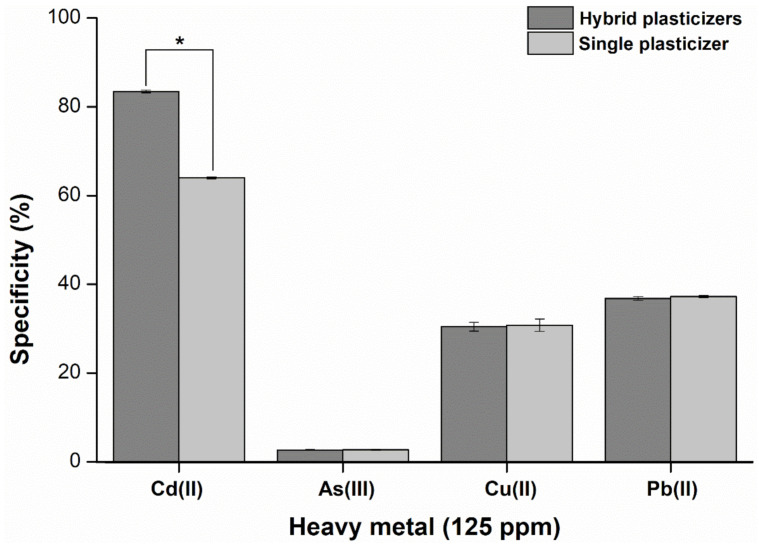
Specificity comparison between the single (DOS) and hybrid (DOS:TOTM) plasticizer sensors composed of TBACl as an ion exchanger (*n* = 5), (* *p*-value < 0.05).

Selectivity is one of the most important factors when determining the reliability of a sensor for practical applications. Therefore, we performed selectivity tests by observing current response for the analysis of cadmium(II) and other heavy metal ions following the procedure from Wang and colleagues [26]. In terms of specificity, the hybrid plasticizer sensor presents a higher specificity value (30%) for cadmium(II) when compared to the single plasticizer sensor (Figure 12). However, the hybrid and single plasticizer sensors demonstrate no significant difference (*p*-value > 0.05) when the two types of sensors are used for detecting As(III), Cu(II), and Pb(II) (Figure 12). These results suggest hybrid plasticizers provide the ability of cadmium ionophore I to bind more strongly with cadmium(II) than is possible in the single plasticizer sensor.

**Figure 13:**
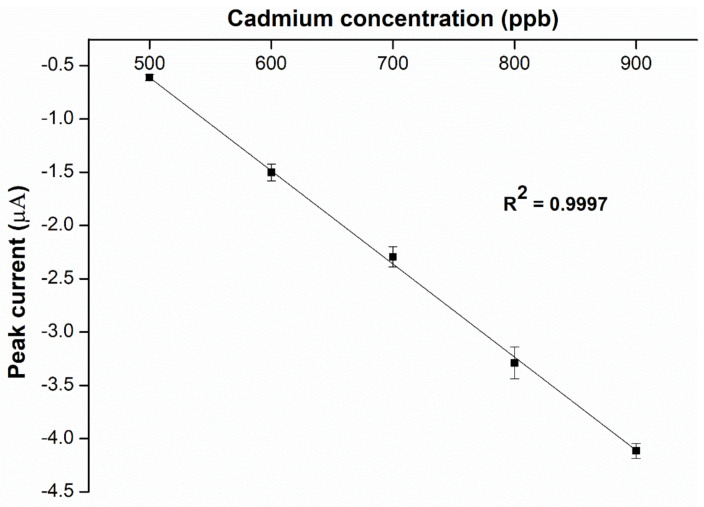
Sensitivity testing of the hybrid plasticizer sensor composed of TBACl at the optimal SE and pH, (*n* = 5). The slope of the graph is 0.00875 μA/ppb.

Cyclic voltammetry is used to determine the sensitivity of our DOS:TOTM (3:1) hybrid cadmium(II) sensor in hydrochloric acid solution of pH 4. The LOD and LOQ values were calculated from the calibration graph of the peak current with a series of cadmium(II) concentrations (Figure 13). The hybrid cadmium(II) sensor achieves results of 95 ppb and 288 for LOD and LOQ, respectively. Compared to previously reported cadmium sensors, the detection limit offered by our DOS:TOTM hybrid sensor is lower than that of other sensors (Table 3). This indicates that the developed hybrid-plasticized sensor is a useful electrochemical sensing platform for cadmium(II) detection.

### 2.8. Determination of Cadmium(II) in Water Samples

The cadmium(II) concentration was successfully measured in spiked water samples by employing the hybrid plasticizer sensor under the optimized conditions. The recovery results (Table 4) revealed that the relative standard deviation (RSD) value was less than 1% for all water samples, indicating excellent recovery rates of close to 100%. It may be assumed that the impurities in the water have no significant influence (*p*-value > 0.05) on the sensor.

## 3. Materials and Methods

### 3.1. Reagents and Samples

Surface cleaning solution (Hellmanex) and heavy metals (cadmium nitrate tetrahydrate, sodium arsenite, lead(II) nitrate, and copper(II) chloride) for cadmium sensor validation were purchased from Sigma-Aldrich (Saint Louis, MO, USA). Chemicals for cadmium-selective membrane construction were tetrahydrofuran (THF), poly(vinyl chloride-co-vinyl acetate-co-vinyl alcohol) (co-PVC), N,N,N′,N′-Tetrabutyl-3,6-dioxaoctanedi(thioamide) (cadmium ionophore I), and plasticizers (2-nitrophenyl octyl ether (NPOE), 2-nitrophenyl phenyl ether (NPPE), dibutyl butylphosphonate (DBBP), 2-fluorophenyl 2-nitrophenyl ether (*f*-NPE), dioctyl phthalate (DOP), diheptyl phthalate (DHP), diphenyl phthalate (DPP), and bis(2-ethylhexyl) sebacate (DOS)) were purchased from Sigma-Aldrich (Saint Louis, MO, USA). The other two plasticizers, tris(2-ethylhexyl) phosphate (TEHP) and tris(2-ethylhexyl) trimellitate (TOTM) were purchased from TCI (Tokyo, Japan). Anion interference test comprises sodium iodide (Merck, Darmstadt, Germany), perchloric acid (Thermo Fisher Scientific, Waltham, MA, USA) and potassium thiocyanate (Thermo Fisher Scientific, Waltham, MA, USA). Tetrabutylammonium hydroxide (TBAOH) for ion exchanger was purchased from TCI (Tokyo, Japan). Ultrapure water used throughout the experiment was purified by a Milli-Q system (Millipore, Merck, Darmstadt, Germany), of which the electrical resistance was 18.2 MΩ·cm.

### 3.2. Surface Cleaning and Membrane Preparation

The surface of SPGE (Wara Microcircuit, Samut Prakan, Thailand) was subjected to 1% *v*/*v* Hellmanex (Hellma, Müllheim, Germany) in water. The cleaned SPGE was immersed in 3 M HCl for 5 min, and dried with argon gas (LabGas, Pathumthani, Thailand). Membrane solution (2.5 µL) was applied on the working electrode of SPGE, and incubated at room temperature for 3 h. The membrane solution was prepared using THF as a solvent (200 µL) for dissolving co-PVC (14.7 mg), plasticizer (44 mg), and cadmium ionophore I (4 mg, 6% *w*/*w*). Then, the membrane-modified SPGE was immersed into a supporting electrolyte. Electropolymerization was performed by using five scans of 10th cycle of cyclic voltammetry with an applied voltage of 1.0 to −1.0 V with Ag/AgCl (3M KCl) at a scan rate of 0.05 V/s.

### 3.3. Voltammetric Measurement

PVC-supported ISE gold working electrode and gold counter electrode of SPGE arranged with Ag/AgCl (3M KCl). Electrochemical cells are as follows:

Ag(s)|AgCl(s)|KCl (3M)||x mM Cd(NO_3_)_2_(aq)|single plasticized PVC|Au(s) (cell 1)

Ag(s)|AgCl(s)|KCl (3M)||x mM Cd(NO_3_)_2_(aq)|hybrid plasticized PVC|Au(s) (cell 2)

Ag(s)|AgCl(s)|KCl (3M)||x mM Cd(NO_3_)_2_ in supporting electrolytes|hybrid plasticized PVC|Au(s) (cell 3)

Ag(s)|AgCl(s)|KCl (3M)||x mM Cd(NO_3_)_2_ in supporting electrolytes|TBACl/hybrid plasticized PVC|Au(s) (cell 4)

Three-electrode setup was employed with a potentiostat (µstat 400, Metrohm, Zofingen, Switzerland), and cyclic voltammogram was observed with the potential scanning within the from 1.0 to −1.0 V at a scan rate of 0.05 V/s. The current response from the sensor was recorded and visualized using DropView 8400 software (Metrohm, Zofingen, Switzerland). The peak currents (µA) could be observed on the cyclic voltammogram used for standard curve analysis.

### 3.4. Semi-Integral Calculation of the Voltammetric Signals

To obtain diffusion coefficients (*D_i_*) and kinetic rate constants of forward reaction (*k_f_*), semi-integral of currents (*I*(*t*)) were calculated based on the recorded voltammetric current response using the following algorithm [24].

(4) $$I\left(t\right)=\sqrt{\frac{\Delta }{\pi }}\overset{t/\Delta }{\underset{j=1}{\sum }}[i\left(j\Delta \right)+i\left(j\Delta -\Delta \right)]\left[\sqrt{\frac{t}{\Delta }-j+1}-\sqrt{\frac{t}{\Delta }-j}\right]$$

The calculation was performed in GNU Octave version 6.4.0 [30] to define values of *I*(*t*).

### 3.5. Selection of a Primary Plasticizer for the Cadmium Sensor

Ten plasticizers (Table 1) were used for the membrane solution optimization. Each membrane solution comprised THF as a solvent (200 µL) for dissolving co-PVC (14.7 mg), plasticizer (44 mg), and cadmium ionophore I (4 mg, 6% *w*/*w*). The optimal plasticizer for the cadmium sensor was determined using cadmium solutions at 25, 50, 75, 100, and 125 ppm.

### 3.6. Selection of Hybrid Plasticizers for the Cadmium Sensor

Types of hybrid plasticizers were optimized to improve the specificity and sensitivity of the cadmium sensor. The hybrid plasticizers were composed of two types of plasticizers, one was the plasticizer from the primary plasticizer experiment (Plasticizer A) combined with another plasticizer (Plasticizer B) (Table 1). Hybrid plasticizers contained 5 to 1 weight ratio of Plasticizer A to Plasticizer B. The cadmium(II)-selective membrane consisted of THF as a solvent (200 µL) for dissolving co-PVC (14.7 mg), total weight of Plasticizer A and B (44 mg), and cadmium ionophore I (4 mg, 6% *w*/*w*).

### 3.7. Optimization for Weight Ratios of the Hybrid Plasticizers

The weight ratios of the hybrid plasticizers (Plasticizer A: Plasticizer B) that yielded the highest current response to the cadmium(II) solution were optimized to improve the sensing sensitivity. Weight ratios of the Plasticizer A to the Plasticizer B were adjusted to 5:1, 3:2, 3:1, 2:1, and 1:1. The membrane thickness was estimated from the weight of membrane compositions on the working electrode and the surface area of electrode (7.07 × 10^−2^ cm^2^) with density of each component [21].

### 3.8. Selection of Supporting Electrolyte (SE) and Influence of pH

SE is an important factor for the sensitivity of the electrochemical sensors, because the resistance value of the solution decreases when mixing an appropriate SE with the analyte [31]. Acetic acid buffer (0.1 M pH 4.5), hydrochloric acid solutions (1.0 mM pH 3, 0.1 mM pH 4, 0.01 mM pH 5) and sodium hydroxide solution (1 µM pH 8) were tested to find the optimal SE in the presence of cadmium(II) at concentrations of 25, 50, 75, 100, and 125 ppm (Cadmium(II) is prepared from cadmium (II) nitrate tetrahydrate). The optimal SE was that which yielded the highest current response, as observed from the voltammogram. The electropolymerization of the optimal membrane, performed with optimal SE, was washed with THF and evaporated in vacuo. The product was characterized by NMR (Bruker, 600 MHz, Billerica, MA, USA) and HPLC-UV (Agilent Technology 1200, Santa Clara, CA, USA). In terms of HPLC separation, the solvent system including water (solvent A) and acetonitrile (solvent B) with the flow rate of 0.600 mL/min was programmed as follows: 0–6 min isocratic elution of 75% solvent B; 6–46 min gradient elution of 0.5%solvent B per minute; 46–56 min isocratic elution of 100% solvent B (column washing); 57–80 min isocratic elution of 75% solvent B (re-equilibration of the column). The HPLC column used in this experiment was a C18 HiChrome analytical column (4.6 mm × 250 mm, 5 μm).

### 3.9. Effect of Scan Rate (ʋ)

To study of the mechanism governing the ion transfer on the electrode surface, cyclic voltammetry was used to determine the ion behavior of the DOS:TOTM hybrid sensor. The solution of 125 ppm cadmium(II) in supporting electrolyte and optimal pH was subjected to different scan rates (0.05, 0.10, 0.20, 0.30 and 0.40 V/s) with a linear-sweep potential from 1.0 to −1.0 V. The plot between peak potential (*E*) and natural logarithm of scan rate (ln ʋ) was performed to determine the mechanism of ion transfer.

### 3.10. Anion Interference

To study the coextraction of anions with cadmium(II) solution of the single plasticizer, the hybrid plasticizer and the hybrid plasticizer composed of tetrabutylammonium chloride (TBACl) sensors, different anions (HClO_4_, KSCN and NaI) were used to study the effect on the current response. TBAOH (1 mM) was used as a ion exchanger for hybrid plasticizer sensor neutralized by hydrochloric acid to obtain TBACl. The detection of current was performed in the presence of anion interferences (0.1 mM each) with 125 ppm cadmium(II) at pH 4 in the supporting electrolyte (0.1 mM HCl) and compared with pristine 0.1 mM HCl.

### 3.11. Specificity and Sensitivity of the Hybrid Plasticizer Sensor

The cadmium sensor and the bare sensor (without the cadmium-selective membrane) were tested with heavy metals including cadmium nitrate tetrahydrate, sodium arsenite, lead (II) nitrate, and copper (II) chloride. Heavy metals were dissolved in the selected supporting electrolyte and optimal pH. The specificity of the sensor was determined according to Wang and colleagues [26]. The limit of detection (LOD) and quantification (LOQ) of the sensor for cadmium(II) can be determined according to the International Council for Harmonisation of Technical Requirements for Pharmaceuticals for Human Use (ICH) [32]. LOD is 3 σ/m and LOQ is 10 σ/m, where σ represents the standard deviation of peak current derived from cadmium(II) in supporting electrolyte, and m is the slope of the current versus cadmium concentration plot [32].

### 3.12. Determination of Cadmium(II) in Water Samples

Water samples (drinking water and tap water) were collected in sterile bottles, and Milli-Q water was used as a control. The pH of the water samples and Milli-Q water were adjusted to the same pH using the selected supporting electrolyte, and cadmium nitrate solution was spiked into the water samples and Milli-Q water at concentrations of 0, 500, and 600 ppb. The %RSD and %recovery were determined according to the ICH guidelines [32].

### 3.13. Statistical Analysis

Peak currents of five replications of all experiments are expressed as mean ± SD. Statistical analysis was performed using IBM SPSS version 18 (IBM Company, Chicago, IL, USA), and the statistical significance level was set to α < 0.05.

## 4. Conclusions

The application of DOS:TOTM-fabricated SPGEs enhanced the analytical performance of the cadmium(II) ion-selective electrode in cyclic voltammetric measurements. The improved analytical performance can be attributed to the optimal flexibility of DOS/TOTM (3:1 *w*/*w*), and the better delocalization of electrons from the gold to the polymer, which induces cadmium(II) to move from the solution to the membrane interface. TBACl can reduce anion interference in the membrane. In addition, the supporting electrolyte (HCl) and pH are also crucial factors playing an important role in the performance of the sensor. To the best of our knowledge, this work is the first to present the concept of hybrid plasticizers, a new paradigm for developing selective membranes used for metal ion detection in order to yield better specificity and sensitivity of the sensing.

## Figures and Tables

**Table 3:** Comparison of the detection limits of cadmium(II) at various modified electrodes.

No.	Electrode	Plasticizer	Ionophore	Response Time (s)	LOD (μM)	Ref.
1	Gold	Hybrid plasticizers (DOS:TOTM)	Cd ionophore I	30	0.8	This work
2	Graphite	Benzyl acetate	1,3-bis(2-cyanobenzene)triazene (L)	2	8.0	[27]
3	Ion selective electrode	DOS	25,27-bis(ethyl-2-(bis(2- pyridylmethyl)aminomethyl)aniline)-26,28-dihydroxy p-tert-butylcalix[4] arene	10	1.6	[28]
4	Graphite	DOS	1,13-bis(8-quinolyl)-1,4,7,10,13-pentaoxatridecane	15	8.4	[29]

**Table 4:** Recovery rate of cadmium(II) ion in spiked water samples.

Sample	Spiked Concentration (ppb)	Found Concentration ^1^ (ppb)	RSD (%)	Recovery (%)	*p*-Value ^2^
Milli-Q water	0	<LOD	-	-	-
	500	498 ± 1.36	0.270	99.6	
	600	592 ± 1.17	0.200	98.7	-
Drinking water	0	<LOD	-	-	-
	500	497 ± 2.20	0.440	99.3	1.000
	600	589 ± 2.51	0.430	98.2	0.362
Tap water	0	<LOD	-	-	-
	500	494 ± 3.47	0.700	98.7	0.231
	600	588 ± 2.78	0.470	97.9	0.146

^1^ Mean ± standard deviation (*n* = 5). ^2^ Significant difference (*p*-value < 0.05).

## Data Availability

Not applicable.

## Supplementary Materials

The following supporting information can be downloaded at: https://www.mdpi.com/article/10.3390/ijms23126402/s1.
