# Supplementary material for: Hybrid Plasticizers Enhance Specificity and Sensitivity of an Electrochemical-Based Sensor for Cadmium Detection
Source: Int J Mol Sci. 2022 Jun 8;23(12):6402. doi: 10.3390/ijms23126402 (PMC9223680; doi:10.3390/ijms23126402)
Supplement: Supplementary file 1 [file ijms-23-06402-s001.zip › ijms-1700314-supplementary.pdf]

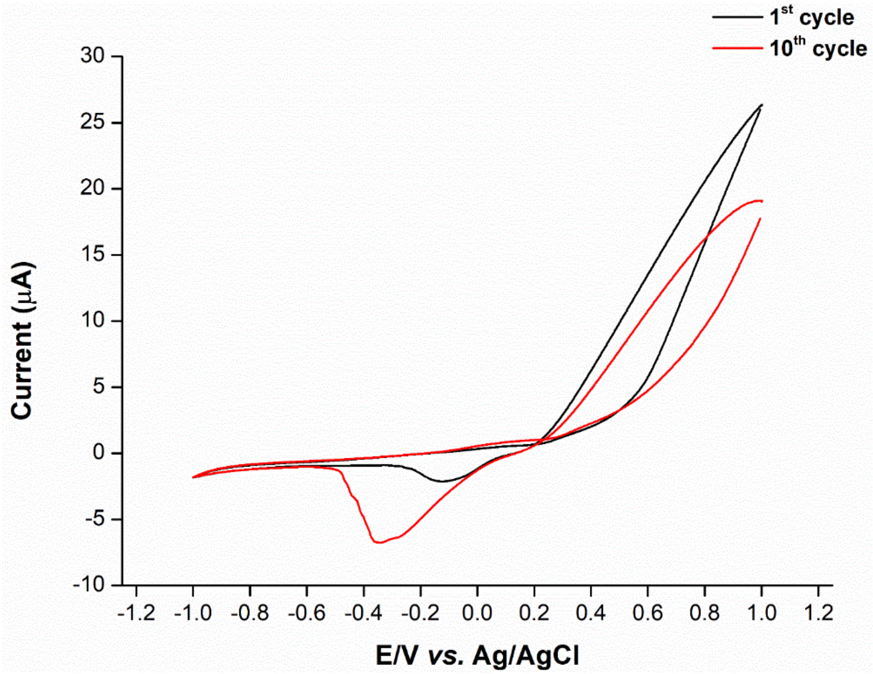

Figure S1. Voltammograms of hybrid plasticizer electropolymerization into co-PVC on gold electrode in 0.1 mM HCl solution at a scan rate 0.05 V/s.

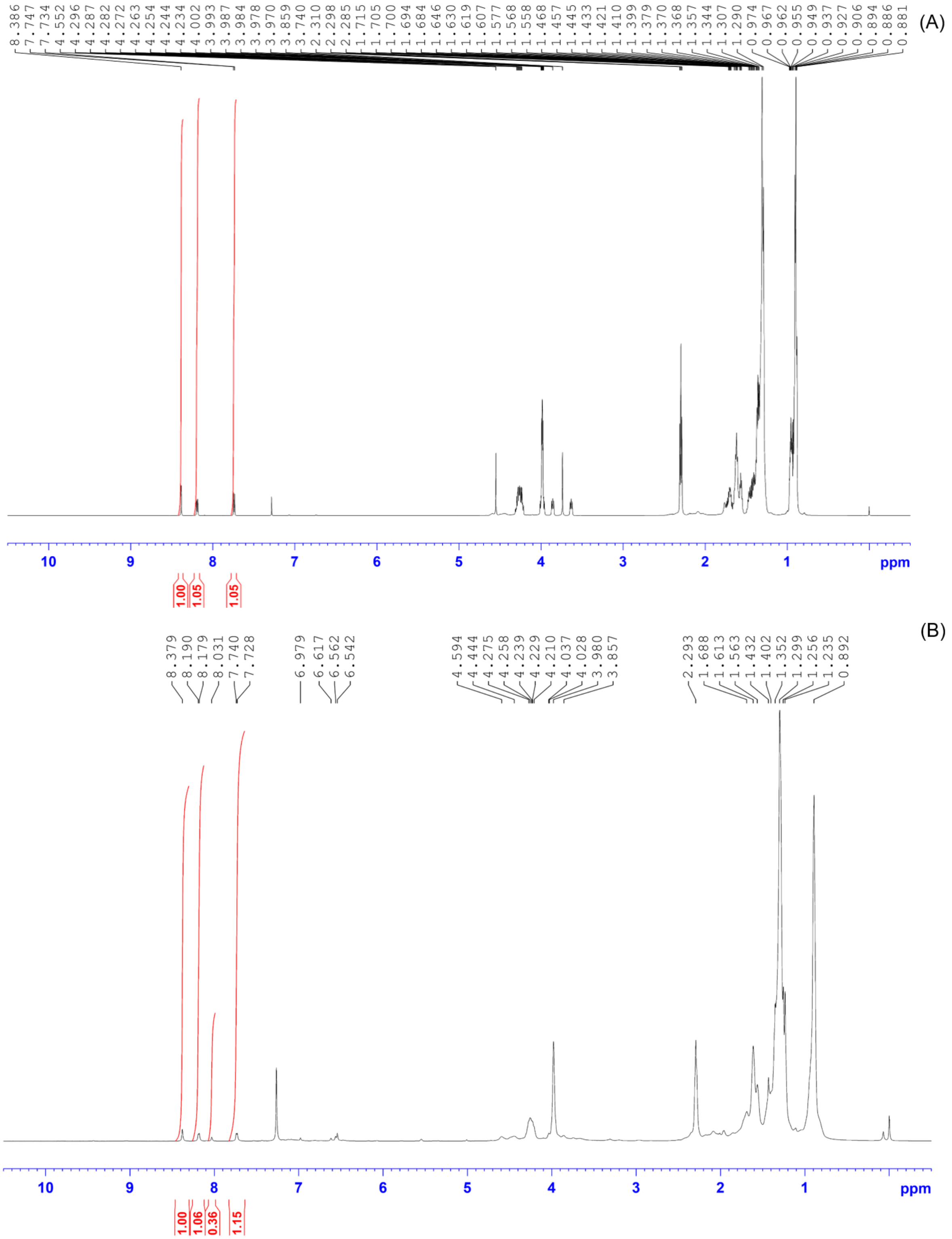

Figure S2. (**A**) 1H-NMR of the starting material mixture. (**B**) 1H-NMR of the mixture after electropolymerization and washed with THF.

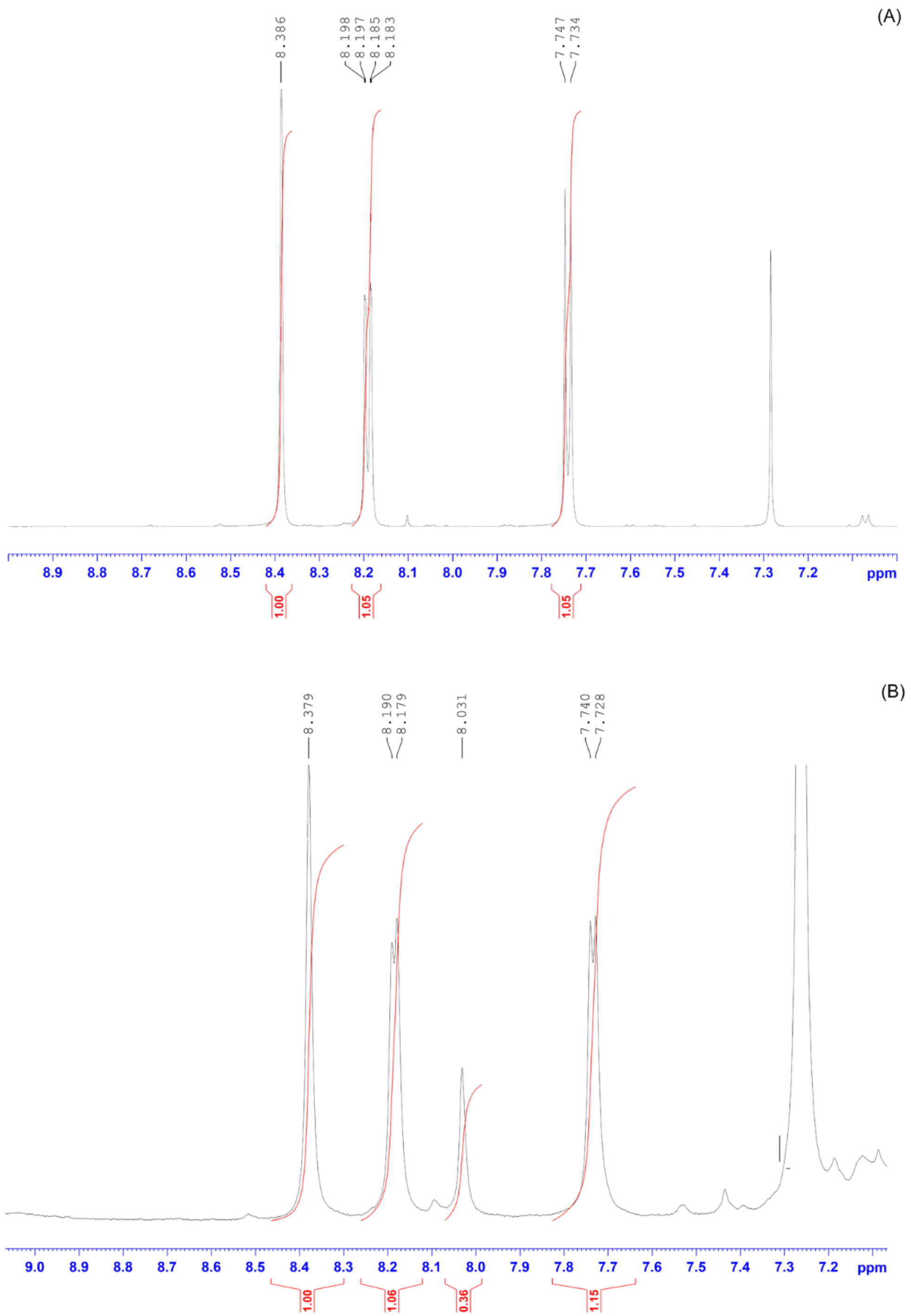

Figure S3. (**A**) The blowup of the chemical shift in the aromatic region of the starting material mixture. (**B**) The blowup of the chemical shift in the aromatic region of the mixture after electropolymerization and washed with THF.

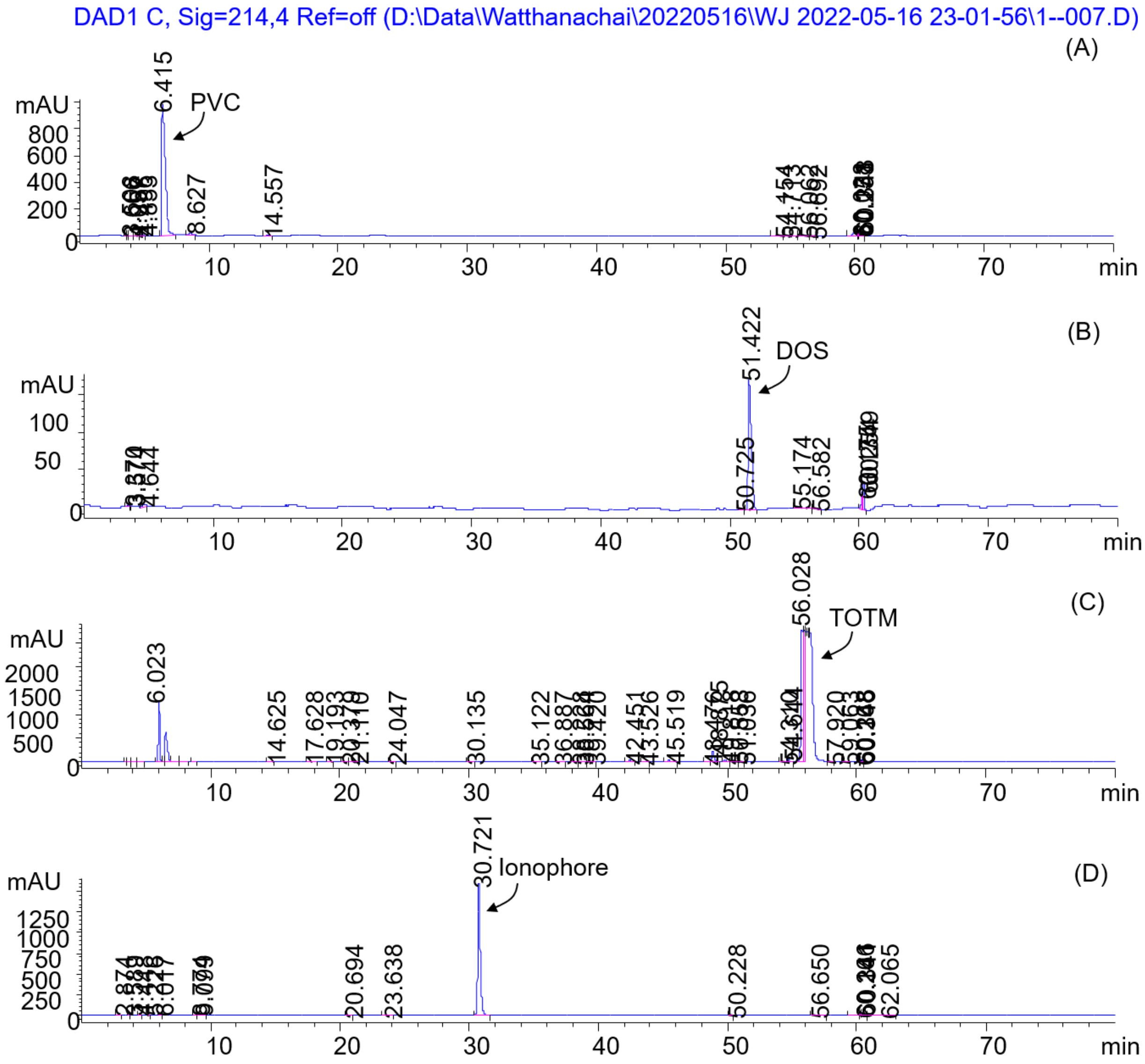

Figure S4. Retention time of the starting materials used for electropolymerization. (**A**) PVC (t_R = 6.4), (**B**) DOS (t_R = 51.4), (**C**) TOTM (t_R = 50.2 min) and (**D**) ionophore (t_R = 30.7 min).

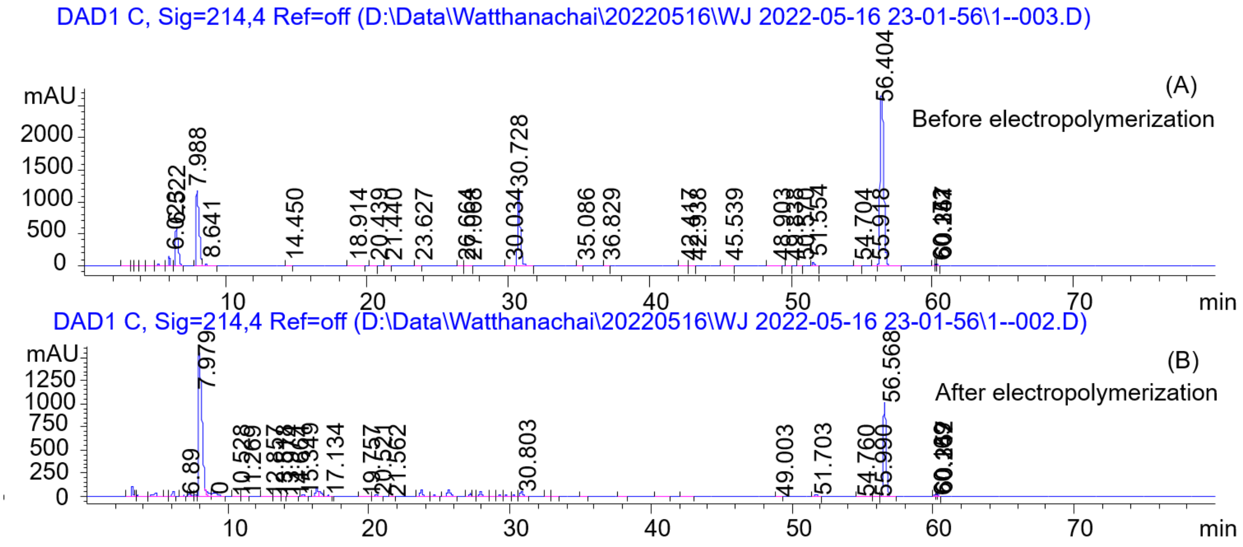

Figure S5. (**A**) Retetion time of the starting material mixture. (**B**) Retention time of the compounds detected by HPLC-UV after electropolymerization and washed with THF.

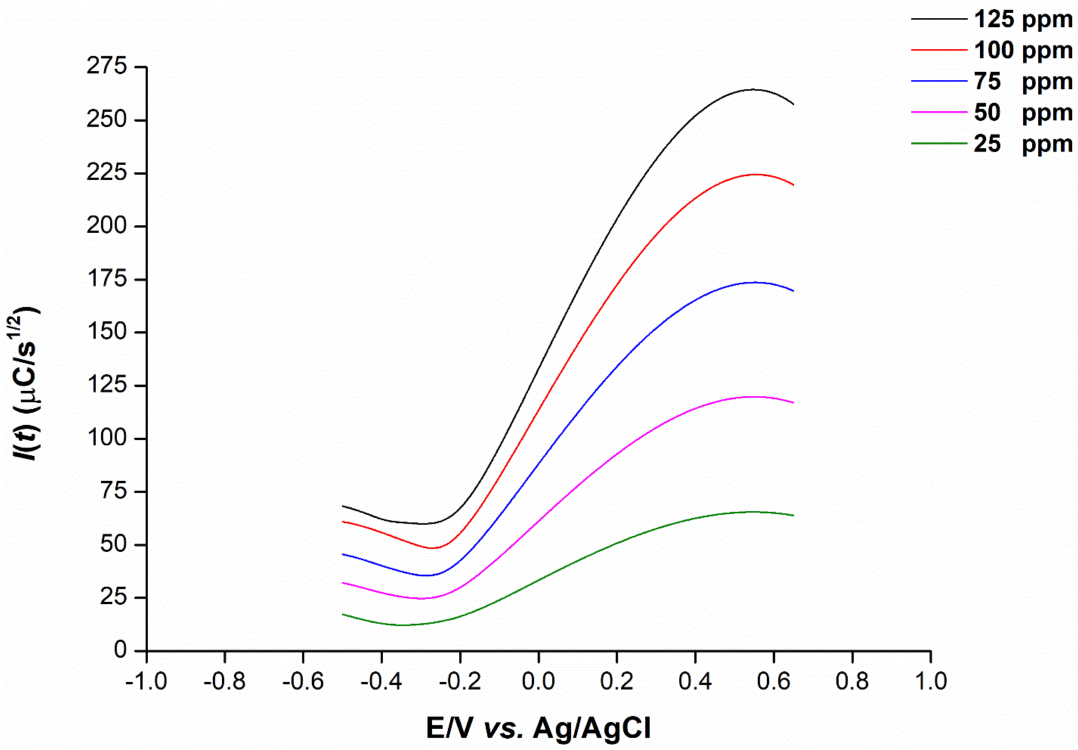

Figure S6. Semi-integral ($I(t)$) of a background-subtracted voltammogram versus potential (E). The voltammogram derived from DOS:TOTM hybrid sensor in 25, 50, 75, 100 and 125 ppm cadmium(II) and 0.1 mM HCl solution at a scan rate 0.05 V/s.

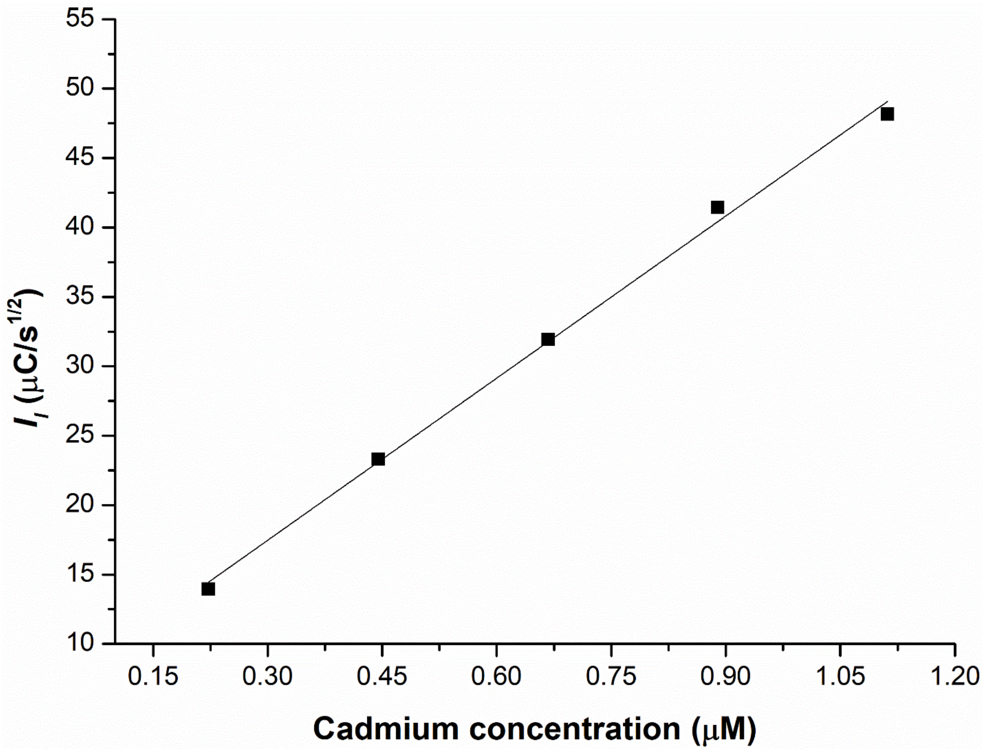

Figure S7. Limiting value of semi-integral (I_l) versus cadmium concentrations in 0.1 mM HCl solution. The slope (I_l/c_i) of linearity is 38.9 C·cm^3·s$^{-1/2}$·mol^{-1} and R-squared as 0.9971. This value was used for calculating diffusion coefficient (D_i) including rate constant of the forward reaction (k_f). Two variables were valid for electrochemical processes of cadmium(II).
